# Desmoglein 2 regulates cardiogenesis by restricting hematopoiesis in the developing murine heart

**DOI:** 10.1038/s41598-021-00996-y

**Published:** 2021-11-04

**Authors:** Hoda Moazzen, Kateryna Venger, Sebastian Kant, Rudolf E. Leube, Claudia A. Krusche

**Affiliations:** Institute of Molecular and Cellular Anatomy, RWTH Aachen University, Wendlingweg 2, 52074 Aachen, Germany

**Keywords:** Differentiation, Transdifferentiation, Haematopoietic stem cells, Embryogenesis, Morphogenesis

## Abstract

Cardiac morphogenesis relies on intricate intercellular signaling. Altered signaling impacts cardiac function and is detrimental to embryonic survival. Here we report an unexpected regulatory role of the desmosomal cell adhesion molecule desmoglein 2 (Dsg2) on murine heart development. A large percentage of *Dsg2*-mutant embryos develop pericardial hemorrhage. Lethal myocardial rupture is occasionally observed, which is not associated with loss of cardiomyocyte contact but with expansion of abnormal, non-myocyte cell clusters within the myocardial wall. Two types of abnormal cell clusters can be distinguished: Type A clusters involve endocard-associated, round-shaped CD31^+^ cells, which proliferate and invade the myocardium. They acquire Runx1- and CD44-positivity indicating a shift towards a hematopoietic phenotype. Type B clusters expand subepicardially and next to type A clusters. They consist primarily of Ter119^+^ erythroid cells with interspersed Runx1^+^/CD44^+^ cells suggesting that they originate from type A cell clusters. The observed pericardial hemorrhage is caused by migration of erythrocytes from type B clusters through the epicardium and rupture of the altered cardiac wall. Finally, evidence is presented that structural defects of Dsg2-depleted cardiomyocytes are primary to the observed pathogenesis. We propose that cardiomyocyte-driven paracrine signaling, which likely involves Notch1, directs subsequent trans-differentiation of endo- and epicardial cells. Together, our observations uncover a hitherto unknown regulatory role of Dsg2 in cardiogenesis.

## Introduction

Desmoglein 2 (Dsg2) is a desmosome-specific transmembrane protein. Its extracellular domain mediates cell–cell adhesion and its cytoplasmic domain associates with desmosomal plaque proteins linking it to the intermediate filament cytoskeleton in epithelial, meningeal and striated cardiac muscle cells^1,2^. In adult heart, Dsg2 functions in desmosome formation and confers structural integrity to the myocardium together with the desmosome-anchored desmin intermediate filament cytoskeleton. Loss or mutation of the desmoglein 2 gene (*Dsg2*) results in arrhythmogenic cardiomyopathy (AC), which is characterized by impaired cardiomyocyte coupling, aseptic inflammation and fibrofatty tissue replacement ultimately leading to heart failure in human patients and animal models^3–10^. The main pathological mechanism of disease initiation is believed to be loss of myocardial adhesion and impaired calcium signaling^4,11,12^.

Dsg2 is also expressed, together with desmosomal plaque proteins, in embryonic cardiomyocytes as early as E10.5 in mice^13,14^. The punctate and desmosome-like expression of Dsg2 changes during heart maturation to a more extended pattern that is typical for the composite intercalated disc encompassing other adherens junctions and gap junctions^13,15^. Despite the early expression of Dsg2 in embryonic hearts, its role in heart development and function has not been investigated.

Evidence has accumulated over the years that functions of Dsg2 extend beyond its contribution to desmosomal adhesion. For example, Dsg2 is expressed in non-desmosome bearing cells such as embryonic stem cells, endothelial progenitor cells and hematopoietic progenitors where it is required for cell proliferation and survival^16–20^. Importantly, Dsg2 has also been recognized as a pluripotent stem cell marker in human embryonic stem cells (hESCs); its loss induces differentiation pathways that facilitate epithelial to mesenchymal transition^17,21^.

We have previously described mice, which carry a mutant *Dsg2* allele lacking exons 4–6 resulting in partial loss of the extracellular domains EC1 and EC2^3^. Homozygous mutant animals develop an AC-like phenotype, which has been proposed to be a consequence of compromised adhesion^3–5,22,23^. In addition, embryonic lethality was noted but not analyzed in detail^3^. We therefore decided to explore the undefined role of Dsg2 in embryonic heart development and function. We observe a pathological emergence of CD31^+^, CD44^+^, Runx1^+^ cell clusters, which differentiate into erythrocytes. The enlarging cell clusters either intercept myocardial connectivity leading to lethal heart rupture or are removed without leaving any permanent damage. Our study highlights an unexpected repressive role of Dsg2 in de novo hematopoiesis in the developing heart involving cardiomyocyte-endocardial cell cross-talk.

## Results

### A large percentage of ***Dsg2***^***mt/mt***^ embryos present pericardial hemorrhage and die during mid gestation

Analyses of embryonic viability revealed a steep increase in mortality among *Dsg2*^*mt/mt*^ embryos during mid gestation. Mortality as evidenced by loss of tissue integrity and cell autolysis rose from 0% at E11.5 to 33.3% at E12.5 and E14.5. Pericardial hemorrhage was frequently found in the remaining vital embryos (*SI Appendix*, Fig. S1; Table S1). Since signs of obvious heart muscle pathology were not detectable by gross visual inspection in these embryos, we decided to examine the mutant hearts by histomorphology. To this end, embryos were age matched with wild-type or heterozygous control littermates, none of which showed any cardiac abnormality. A summary of the observations described in the following sections is presented in Table S1.

### Abnormal endocard-associated type A and subepicardial type B cell clusters develop in the heart of *Dsg2*^*mt/mt*^ embryos

**Figure 1:**
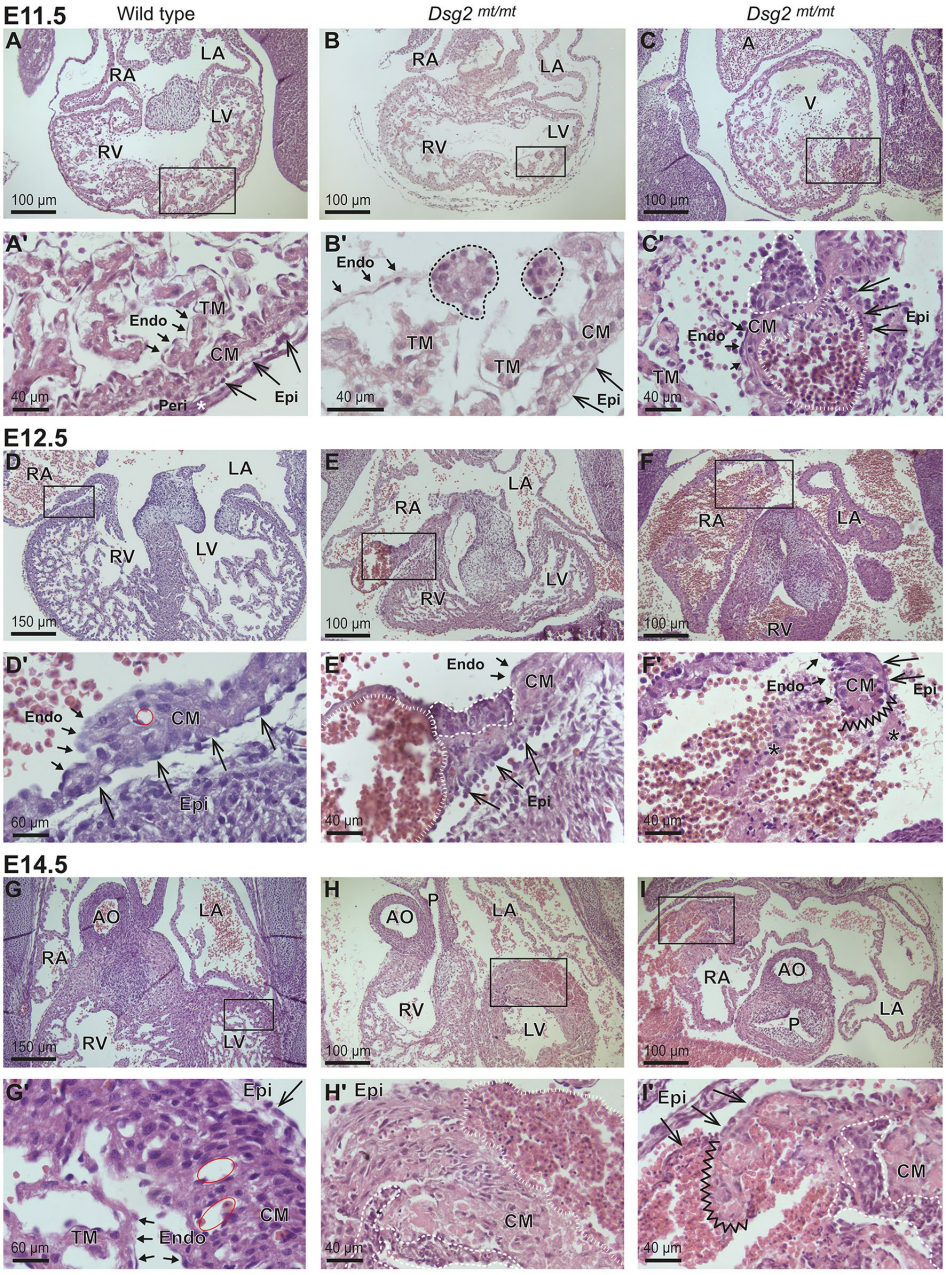
Histological comparison of wild-type and *Dsg2*^*mt/mt*^ embryonic hearts reveals two types of pathological cell clusters and rupture in the mutants. (**A**-**I**ʹ) The images show photomicrographs of hematoxylin/eosin-stained heart sections at embryonic days E11.5, E12.5 and E14.5 at low and high magnification. The boxed areas in the upper rows correspond to the magnified regions below. Prominent structures, regions and cell layers are labeled: RV, right ventricle; LV, left ventricle; RA, right atrium; LA, left atrium; A, atrium; V, ventricle; AO, aorta; P, pulmonary artery; Endo, endocardium (short arrows); Epi, epicardium (long arrows); Peri, pericardium; CM, compact myocardium; TM, trabecular myocardium. (**A**,**A**ʹ,**D**,**D**ʹ,**G**,**G**ʹ) exemplify wild-type heart morphology at the different days of gestation. Note the appearance of capillaries at E12.5, which become more numerous and enlarged by E14.5 (red circles). (**B**–**B**ʹ) The dashed lines delineate two abnormal type A cell clusters in *Dsg2*^*mt/mt*^ hearts consisting of densely packed cells that are in continuity with the endocardium. (**C**–**C**ʹ) depicts a type A cell cluster (dashed line) next to a type B cell cluster (striated line) containing numerous nucleated erythrocytes in the subepicardium of a *Dsg2*^*mt/mt*^ heart. (**E**–**E**ʹ) shows an expanded type B erythrocyte cell cluster (striated line; the empty space is caused by cell loss during tissue processing) in the subepicardium of the right atrium at E12.5 that is directly next to a type A cell cluster (dashed line). (**F**–**F**ʹ) The pictures depict a rupture (marked by zig-zag line) in the right atrium of a *Dsg2*^*mt/mt*^ heart. Asterisk in (**F**ʹ) marks remaining cells of the ruptured myocardium. (**H**–**H**ʹ) the picture pair shows a large type B cell cluster (striated line) and a type A cell cluster (dashed line) in the left ventricle. (**I**–**I**ʹ) depict a rupture (marked by zigzag line) in the right atrium of a *Dsg2*^*mt/mt*^ heart. An adjacent type A cell cluster is delineated by a dashed line. Images are representative of N = 16 at E11.5, N = 9 at E12.5 and N = 8 at E14.5 in wild-type and heterozygous control groups and N = 24 at E11.5, N = 12 at E12.5 and N = 12 at E14.5 for the *Dsg2*^*mt/mt*^ group. Size bars: 100 μm in (**A**,**B**,**C**,**E**,**F**,**H**,**I**); 150 μm in (**D**,**G**); 40 μm in (**A**ʹ,**B**ʹ,**C**ʹ,**E**ʹ,**F**ʹ,**H**ʹ,**I**ʹ) and 60 μm in (**D**ʹ,**G**ʹ).

**Figure 2:**
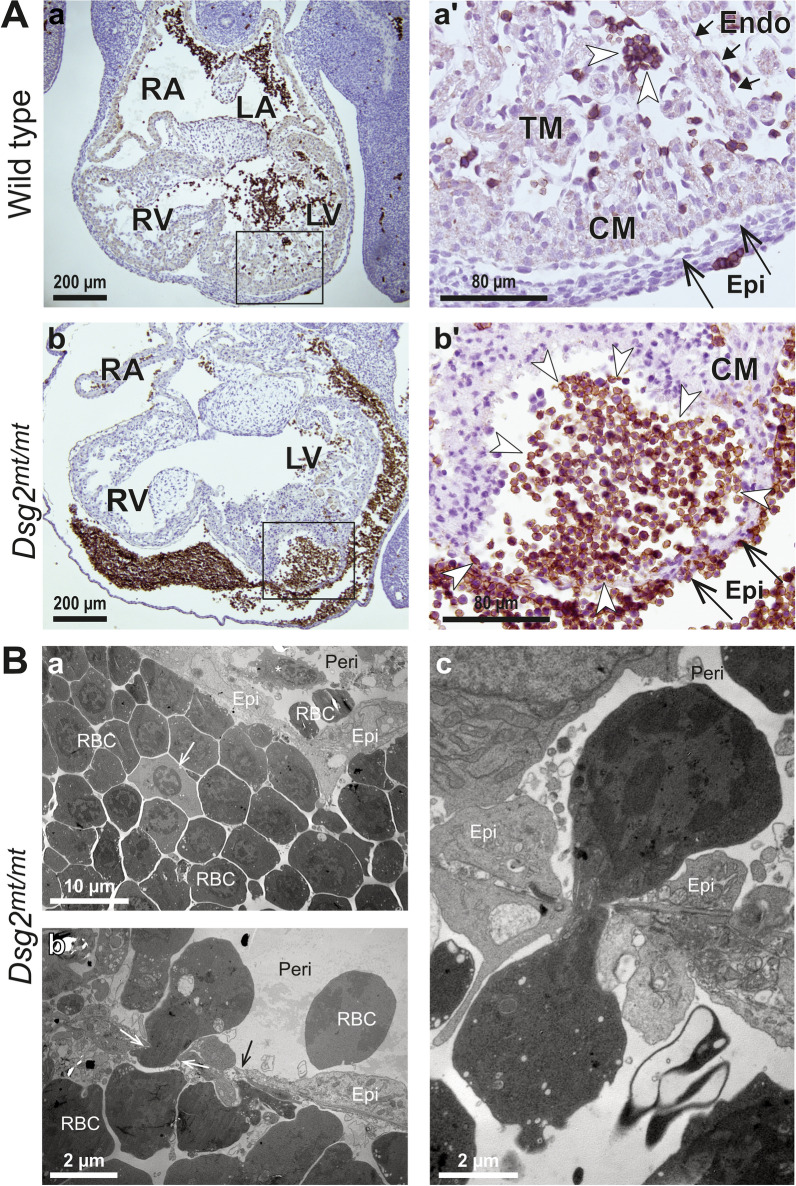
Expansion of erythrocytes in *Dsg2*^*mt/mt*^ hearts. (**A**) Immunohistochemical Ter119 staining shows that type B cell clusters in *Dsg2*^*mt/mt*^ hearts consist of differentiating erythrocytes (n = 3 hearts per group). The picture pairs depict E11.5 heart sections prepared from a wild-type (**a**–**a**ʹ) and a *Dsg2*^*mt/mt*^ embryo (**b**–**b**ʹ) at low and higher magnification (boxed areas). Note that only circulating erythrocytes are Ter119^+^ in the wild-type heart (brown; white arrowheads in **a**ʹ) whereas a large subepicardial accumulation of abnormal cells is strongly Ter119^+^ in the *Dsg2*^*mt/mt*^ heart (white arrowheads in **b**ʹ). The mutant heart also contains aberrant Ter119^+^ cells in the pericardial space. RA, right atrium; LA, left atrium; RV, right ventricle; LV, left ventricle; Endo, endocardium (short arrows); Epi, epicardium (long arrows). Size bars: 200 µm in (**a,b**); 80 µm in (**a**ʹ,**b**ʹ). (**B**) Electron microscopy reveals that differentiating erythrocytes migrate into the pericardial cavity in the heart of an E11.5 *Dsg2*^*mt/mt*^ embryo. (**a**) Shows red blood cells (RBC) surrounding an undifferentiated pale cell (arrow) in a type B cell cluster that is directly adjacent to the epicardial cell layer (Epi). Note that a migrating cell (*) and red blood cells (RBC) are detected in the pericardial space (Peri). (**b**,**c**) The higher magnification images show aspects of red blood cell migration through the epicardium into the pericardial space (same abbreviations as in **a**). Note, how red blood cells squeeze through narrow gaps (e.g., white arrows in **b**). The black arrow demarcates a thinned epicardial cell that will likely be penetrated by the adjacent deformed red blood cell. Size bars: 10 μm in (**a**), 2 μm in (**b**,**c**).

In E11.5 wild-type embryonic hearts, several layers of cardiomyocytes are covered by flat endocardium facing the inner lumen of the heart chambers and by flat epicardium facing the pericardial cavity. The embryonic ventricular cardiomyocytes are divided into outer, round-shaped, proliferative cardiomyocytes forming the compact myocardium and inner, elongated, more differentiated cardiomyocytes forming the protruding trabecula (Fig. 1A,Aʹ). In contrast to all 16 wild-type E11.5 embryonic hearts, 18 of 24 serially sectioned E11.5 *Dsg2*^*mt/mt*^ hearts presented atypical cell clusters (Fig. 1B–Cʹ). Two types of cell clusters could be distinguished. Type A clusters consisted of densely packed cells that formed nodular structures next to the endocardium (Fig. 1B,Bʹ). In most instances, multiple such cell clusters were observed at different locations with a predilection for the endocardium lining the trabecular myocardium. The cell clusters were of variable size and consisted of homogeneous cell populations that differed from the adjacent tissues. In proximity to the type A cell clusters accumulations of erythroid cells were detected within the compact myocardium layer and next to the subepicardium of some mutant embryos. We will refer to them as type B cell clusters (Fig. 1C,Cʹ). The erythroid cells of the type B clusters were found to be positive for Ter119, which is expressed in all stages of red blood cell differentiation from early proerythroblasts to mature erythrocytes (Fig. 2A). The type B clusters were massive and much larger than the minute islands that have been described in the subepicardium of the wild type^24^. Such clusters were not detected in any of the wild-type control hearts. The type B cell clusters were not demarcated by an endothelium but were directly adjacent to epicardium and cardiomyocytes. The adjacent cardiomyocytes were stretched, which appeared to be caused by cluster expansion. Myocardial trabeculation was considerably reduced adjacent to both type A and type B cell clusters. In addition, accumulation of different amounts of blood was detected in the pericardial cavity in the vast majority of abnormal cases (83.3%). The source of the blood, however, could not be identified and the myocardial wall appeared to be intact. Anti-Ki67 immunostaining further revealed that both types of cell clusters consisted of proliferating cells (*SI Appendix*, Fig. S2). Immunostaining for cleaved caspase-3 showed that myocardial cell death does not occur in the myocardium at regions next to expanding clusters (*SI Appendix*, Fig. S3).

Twelve E12.5 Dsg2^mt/mt^ mutant hearts were then compared to 9 heterozygous control hearts. The E12.5 hearts were considerably larger than those of E11.5 embryos and presented increased myocardial wall thickness and starting capillary formation (Fig. 1D,Dʹ). One third of the E12.5 *Dsg2*^*mt/mt*^ embryos were dead at the time of harvest, and the majority of the remaining vital embryos showed abnormal heart morphology (75%). Similar to the E11.5 mutant embryos, type A and type B cell clusters were detected in E12.5 mutant embryos (Fig. 1E–Eʹ). Typically, both lesion types were found close to each other (e.g., Fig. 1Eʹ). The percentage of hearts with type A and B cell clusters, however, was lower at this embryonic stage compared to E11.5. A possible explanation is that E11.5 embryos with extensive cell clusters had died. This is in line with the observed increased number of resorbed embryos at E12.5. Furthermore, we were able to document a massive heart rupture in the atrium of one embryo (Fig. 1F,Fʹ).

The histology of 12 E14.5 *Dsg2*^*mt/mt*^ mutants was then compared to that of 8 heterozygous control embryos. At this time, ventricles were partially separated by a septum, and a network of blood vessels had formed in the myocardium (Fig. 1G,Gʹ). Abnormal subepidcardial type B cell clusters were frequently observed with a relative increase compared to E11.5 and E12.5. We did not find any indication that the clusters of developing erythrocytes were connected to developing blood vessels. Pericardial blood was still frequently seen in alive-harvested mutants (87.5%) and myocardial rupture was detected in ventricles or atria of 50% of the harvested E14.5 embryos (Fig. 1I,Iʹ). Each of the ruptures involved expanded type B lesions, which released the developing erythrocytes into the pericardial cavity and the lumen of the heart chambers. Of note, type A cell clusters were always found next to expanded erythrocyte colonies (Fig. 1C,E,H,I).

### Abnormal cell clusters are demarcated from adjacent cardiomyocytes in *Dsg2*^*mt/mt*^ embryos

**Figure 3:**
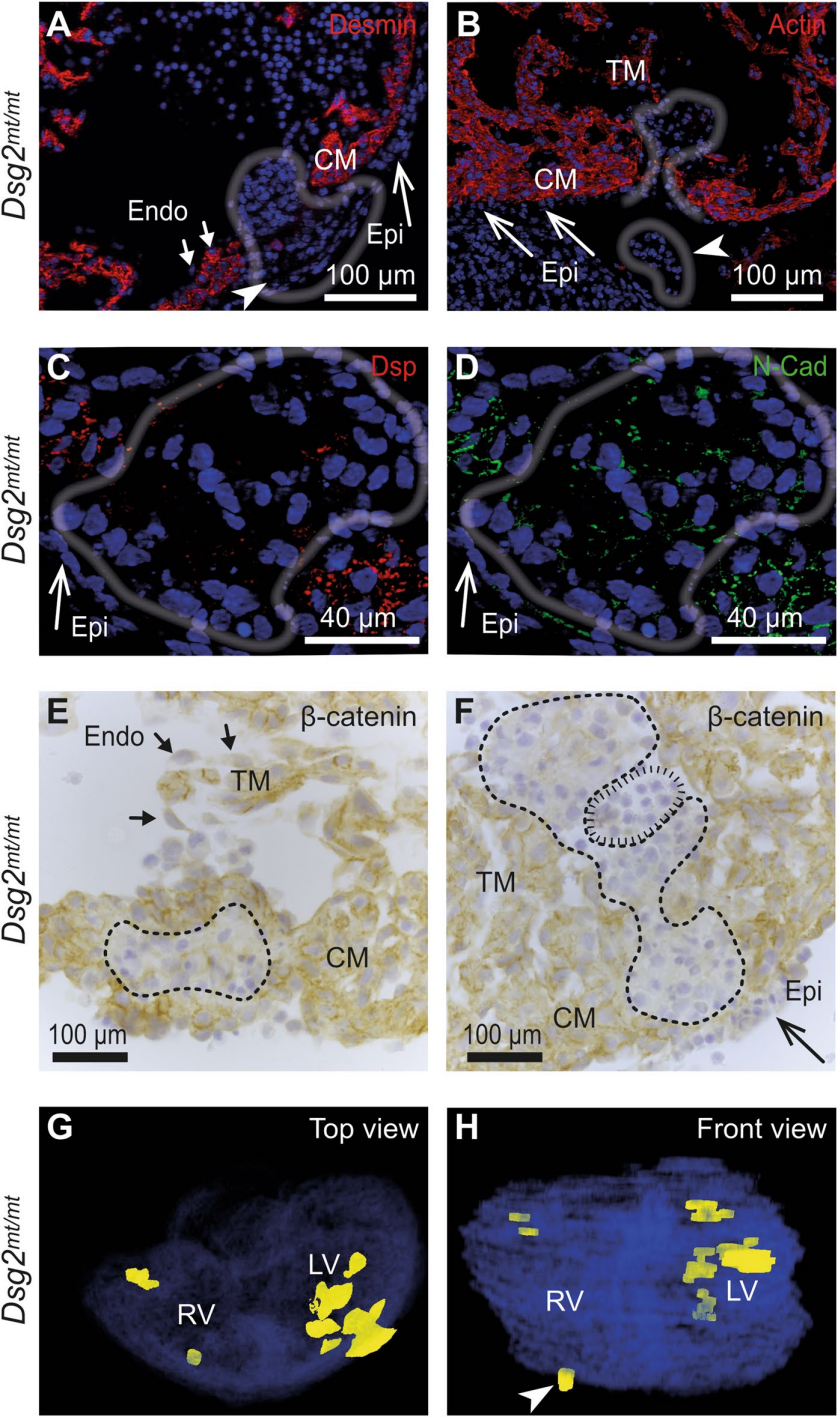
Abnormal cell clusters are for the most part negative for cardiomyocyte markers. (**A**,**B**) The E11.5 *Dsg2*^*mt/mt*^ heart sections are stained with anti-desmin or anti-fetal cardiac actin antibodies revealing strictly demarcated negative cell clusters (gray contour lines). Note the large number of nuclei in the pericardial cavity (arrowheads) and the extrusion of a compact cell cluster (arrowhead and delineated by gray contour line in **B**). CM, compact myocardium; Epi, epicardium (long arrows); Endo, endocardium (short arrows). (**C**–**F**) show immunohistological micrographs of *Dsg2*^*mt/mt*^ heart sections containing cell clusters that express little or no desmoplakin (Dsp), present reduced N-cadherin (N-Cad) and are negative for β-catenin. Contour lines delineate the entire cell clusters in the consecutive sections in (**C**,**D**), dashed lines demarcate type A clusters in (**E**,**F**) and the striated line in (**F**) encircles a small type B cell cluster. TM, trabecular myocardium; (**G**,**H**) Top and front views of a 3D reconstruction prepared from 40 serial hematoxylin/eosin-stained sections of an E11.5 *Dsg2*^*mt/mt*^ heart. The abnormal cell clusters are colored in yellow and ventricular myocardium in blue. RV, right ventricle; LV, left ventricle. The white arrowhead points to a cluster of cells that is released into the pericardial cavity. Data are representative images of n = 5–6 hearts per group and analysis. Size bars: 100 μm in (**A**,**B**,**E**,**F**) and 40 μm in (**C**,**D**).

To characterize the relationship between the type A and type B cell clusters and the surrounding myocardium, we performed immunostaining with cardiomyocyte-specific markers. Both cell clusters did not express detectable amounts of desmin or fetal cardiac actin (Fig. 3A,B). Very minor and locally restricted immunoreactivity was detectable for the desmosomal plaque protein desmoplakin (Fig. 3C). Reduced immunoreactivity was observed for the adherens junction protein N-cadherin whereas expression of the adherens junction protein β-catenin was undetectable in the cell clusters (Fig. 3D–F). Taken together, we concluded that the abnormal cell clusters have a non-cardiomyocyte identity.

### Abnormal cell clusters are extruded from the myocardium or release erythrocytes into the pericardial cavity in *Dsg2*^*mt/mt*^ embryos

3D reconstruction of serial histological sections revealed that the intramural cell clusters formed structures with diameters up to several 100 µm (Fig. 3G,H). The cell clusters were preferentially localized in the right atrium or left ventricle. Strikingly, some of the larger cell clusters encompassed the entire myocardial wall protruding to both the lumen of the heart chambers and pericardial cavity. In several instances, circumscribed cell clusters were identified in the pericardial cavity. They were either completely detached from the myocardium or were in loose connection with the epicardium (Fig. 3B,E–F). These observations suggested that extrusion is a mechanism of cell cluster removal. Another mechanism of cell cluster resorption was observed in electron micrographs (Fig. 2B). They revealed that multiple nucleated erythrocytes breached the epicardial cell layer by transmigration. This process is most likely the reason for the high percentage of pericardial blood accumulation in the absence of visible rupture. Both, the extrusion of cell clusters from the myocardium and the transmigration of nucleated erythrocytes into the blood stream or the pericardial cavity may explain, why surviving mutant embryos develop into full-term newborns with normal-appearing myocardium^3^.

### Endo- and epicardium may contribute to the formation of subendocardial type B cell clusters of *Dsg2*^*mt/mt*^ embryos

**Figure 4:**
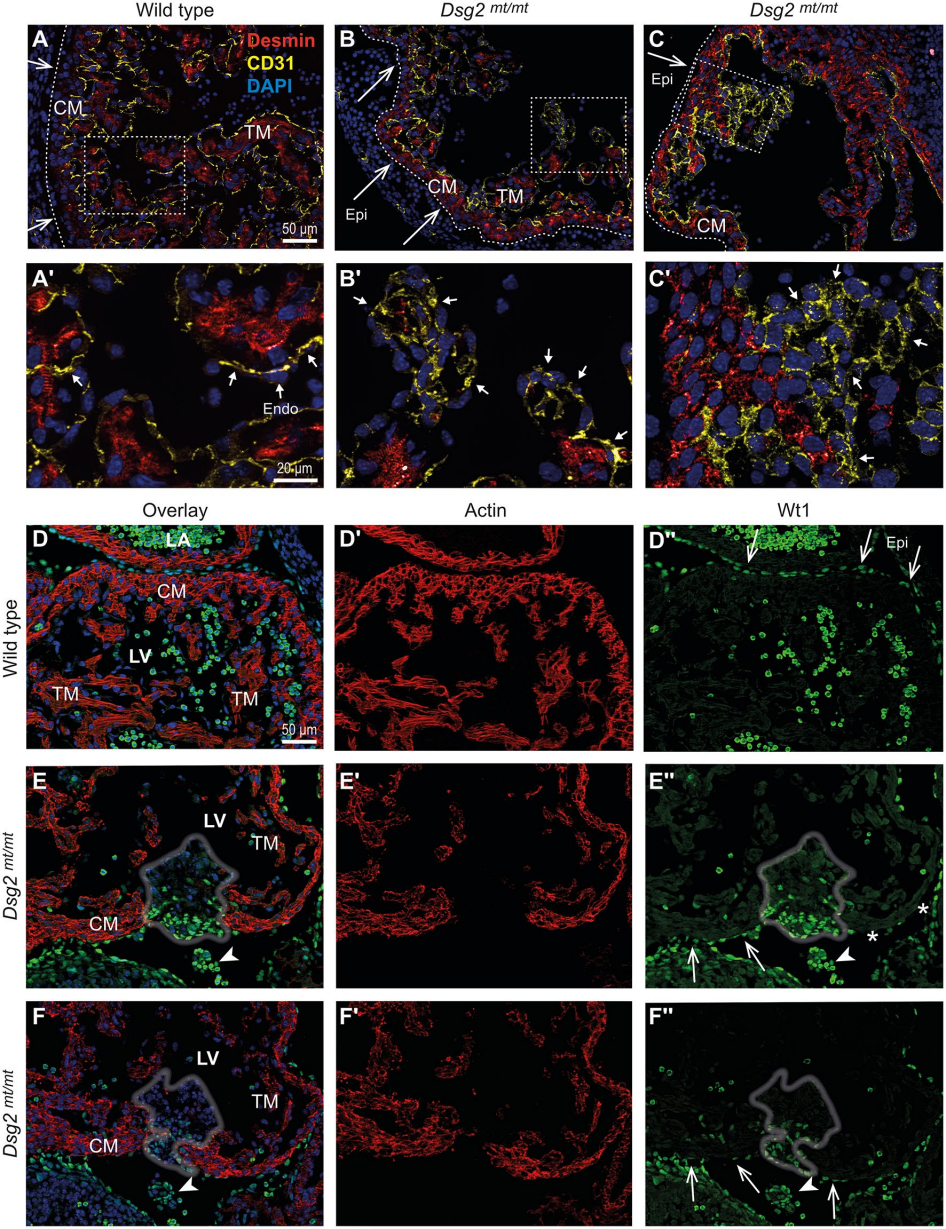
Endothelial CD31^+^ and epicardial Wt1^+^ cells expand in E11.5 *Dsg2*^*mt/mt*^ hearts. (**A**–**C**ʹ) The immunofluorescence micrographs detect desmin (red), CD31 (yellow) and nuclei (DAPI, blue) in wild-type and *Dsg2*^*mt/mt*^ hearts (high magnifications in **A**ʹ–**C**ʹ depict regions boxed in **A**–**C**). Long arrows point to the epicardium (Epi; dashed lines), short arrows to the endocardium (Endo). In addition to CD31^+^ endothelial cells, CD31^+^ cell clusters are visible in the ventricle and atrium of *Dsg2*^*mt/mt*^ hearts in (**B**ʹ) and (**C**ʹ), respectively. Note the reduction of trabecular myocardium (TM) in (**B**) compared to (**A**). CM, compact myocardium. (**D**–**F**ʺ) Immunostaining of embryonic heart sections detects fetal cardiac actin (red), Wt1 (green) and nuclei (blue) in wild-type and *Dsg2*^*mt/mt*^ hearts. Note that the nucleated blood cells circulating in the heart chambers show strong green cytoplasmic autofluorescence, which differs from the nuclear Wt1 signal (long arrows in **D**ʺ). The non-cardiomyocyte cell clusters are marked by gray contour lines in (**E**,**E**ʺ,**F**,**F**ʺ). An increased number of atypical Wt1^+^ cells are detected on the epicardial side of the cell clusters and a few scattered Wt1^+^ cells are also present inside the clusters. Note also, that the myocardium of *Dsg2*^*mt/mt*^ hearts shows regions without epicardial Wt1^+^ cells (asterisk in **E**ʺ). Data are representative images of n = 5–6 hearts per group and analysis. Size bars, 50 µm in (**A**) (same magnification in **B**, **C**); 20 µm in **A**ʹ (same magnification in **B**ʹ, **C**ʹ); 50 µm in (**D**) (same magnification in **D**ʹ–**F**ʺ).

Since endothelial endocardium encompasses a niche of putative hematopoietic stem cells (hemogenic endothelium) on the trabecular myocardial extensions^24,25^, we hypothesized that the abnormal type A cell clusters may have endocardial cell identity. Thus, expansion of endocardial cells with hemogenic potential might primarily form type A cell clusters and subsequently differentiate into the erythroid cells of type B cell clusters. To examine this possibility, we stained tissue sections with antibodies against the endothelial marker CD31. As expected, CD31-positive, endocardial cells were detectable in E11.5 wild-type hearts (Fig. 4A,Aʹ). They formed a single layer of flat endothelial cells lining the lumen of the heart chamber. Although endocardial endothelial cells were also CD31^+^ in *Dsg2*^*mt/mt*^ hearts, many of them were not flat but round-shaped and formed multiple layers decorating a myocardium with considerably reduced trabeculation. Most remarkably, the majority of type A cell clusters were CD31^+^ (Fig. 4B–C').

On the other hand, hematopoietic capacity is also ascribed to the epicardium during embryogenesis^26^. Anti-Wt1 antibodies were used to detect epicardial cells. As expected, Wt1^+^ epicardial cells were detected covering the myocardial surface as a single layer in wild-type and most regions of the mutant myocardium (Fig. 4D–Fʺ). The *Dsg2*^*mt/mt*^ epicardium appeared to be unaffected in regions where the type A cell clusters were restricted to the endocardial side of the cardiac wall. However, Wt1^+^ epicardial cells formed multiple layers adjacent to the type A and B cell clusters that had expanded within the ventricular wall (Fig. 4E,Eʺ). Most of these additional Wt1^+^ cells lost their flat morphology and obtained a round shape. Occasionally, these cells were identified circulating in the pericardial cavity and in connection with the abnormal cell clusters (arrowheads in Fig. 4E,Eʺ,F,Fʺ). Co-localization of Wt1 and cardiac actin was not observed (Fig. 4D–Fʺ). Together, the findings indicate that both epicardial and endocardial cells may contribute to the formation of abnormal red blood cell clusters in *Dsg2*^*mt/mt*^ hearts through distinct intermediaries.

### Runx1^+^ and CD44^+^ cells expand predominantly in endocardium-associated type A cell clusters of *Dsg2*^*mt/mt*^ hearts

**Figure 5:**
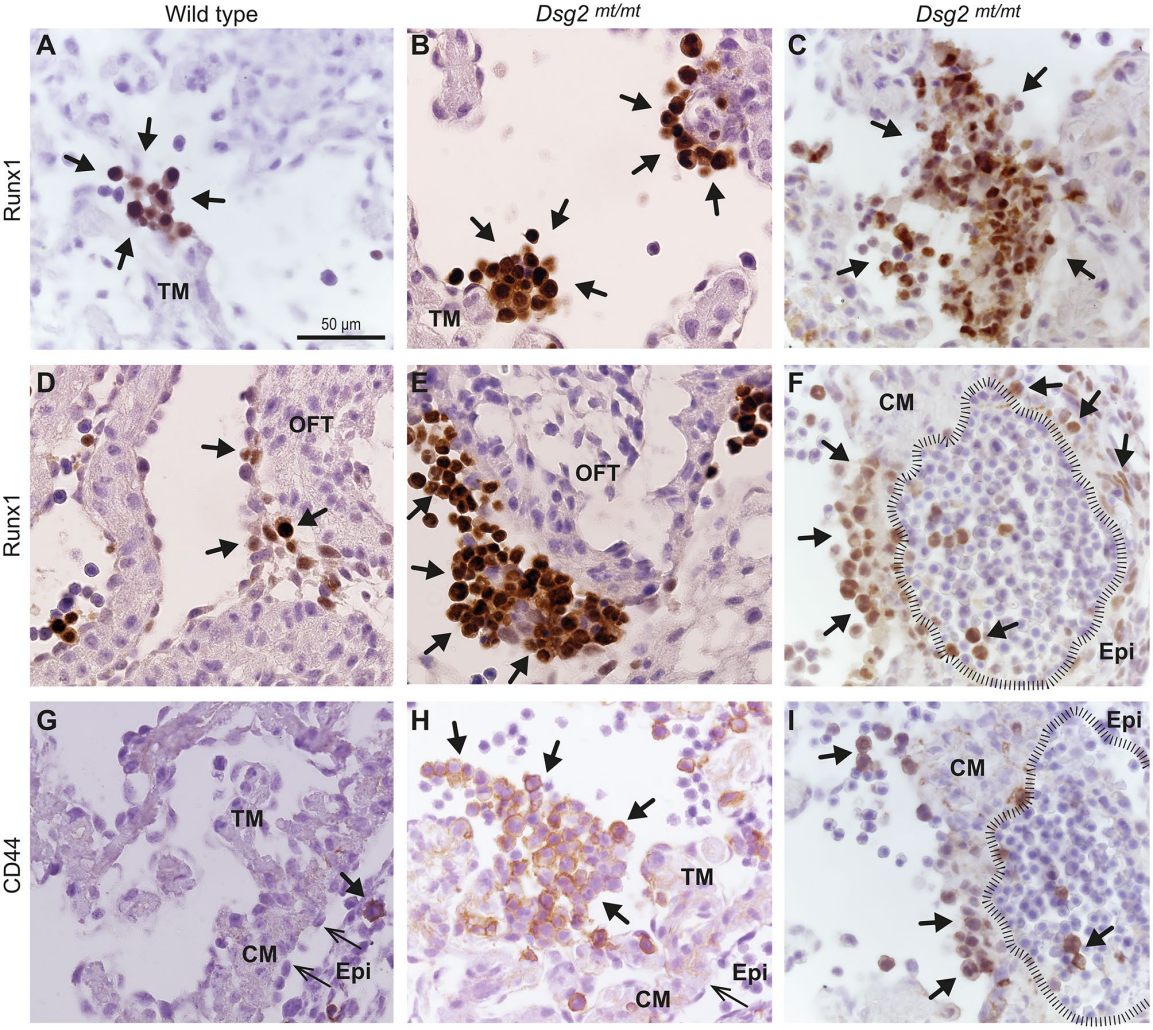
Hematopoietic stem and progenitor cells (HSPCs) expand in non-ruptured hearts of *Dsg2*^*mt/mt*^ embryos at E11.5. (**A**) Short arrows point to rarely detected nuclear Runx1 expression (brown) at the tip of trabecular myocardium (TM, arrows) in wild-type hearts. (**B**–**C**) Differently sized clusters of Runx1^+^ cells (short arrows) are depicted in the left ventricle protruding into the chamber lumen of *Dsg2*^*mt/mt*^ hearts. (**D**,**E**) Runx1^+^ cells (short arrows) are sparse in epi- and subepicardium of the outflow tract (OFT) in wild-type and abundant in the *Dsg2*^*mt/mt*^ heart. (**F**) depicts Runx1 staining in a type A cell cluster (short arrows on the left side of image) next to a type B cell cluster (striated line) and an abnormal epicardial bulge containing some Runx1^+^ cells. Note the presence of single Runx1^+^ cells within the differentiating and proliferating erythrocyte colonies. CM, compact myocardium. (**G**) shows a wild-type heart section immunostained for CD44. Arrow points to a single round-shaped cell in capillary (short arrow). Epi, epicardium (long arrows); CM, compact cardiomyocytes (**H**) CD44^+^ cells are detected in a type A cell cluster in the left ventricle of a *Dsg2*^*mt/mt*^ heart. (**I**) Single CD44^+^ cells are also detected next to type B cell cluster (striated line; serial section to **F**). Data are representative images of n = 4–5 hearts per group and analysis. Size bar, 50 µm in (**A**) (same magnification in all other pictures).

To further dissect the derivation of the erythroid cells in the type B cell clusters, we stained cardiac tissue sections with antibodies against Runx1, which is a well-established marker of definitive hematopoiesis^27–29^. In wild-type E11.5 hearts some Runx1^+^ cells were observed in the blood. In rare instances, small clusters of Runx1^+^ cells were detected that were either associated with the endocardium or the epicardium of the outflow tract (Fig. 5A,D). Much in contrast, multiple Runx1^+^ type A cell clusters of different size were seen in *Dsg2*^*mt/mt*^ hearts (Fig. 5B,C). Runx1^+^ cells were also abundantly expanded in the epicardium of the outflow tract, which is connected to the ventricular chamber (Fig. 5E).

Next, we examined Runx1 expression in type B cell clusters of E11.5 hearts. The large type B cell cluster in the left ventricle depicted in Fig. 5F contained few Runx1^+^ cells that were surrounded by Runx1^−^ erythrocytes. In contrast, the majority of cells in the adjacent type A cell cluster were Runx1^+^. Remarkably, the type A and type B cell clusters were not separated by cardiomyocytes indicating that cellular exchange may take place between both. Occasionally, single Runx1^+^ cells were also seen in regions of expanded epicardial cells (Fig. 5F).

We then studied the expression of CD44, which is an early marker and regulator of endothelial to hematopoietic transition^30^. CD44^+^ cells were almost absent in the wild-type endocardium, myocardium and epicardium of E11.5 embryos and were only rarely detectable in blood (Fig. 5G). In contrast, CD44^+^ cells were found to be in the majority of type A cell clusters and single CD44^+^ cells were interspersed in type B cell clusters of the *Dsg2*^*mt/mt*^ hearts (Fig. 5H,I). By double immunofluorescence microscopy, we were also able to identify a few atypical round-shaped Wt1^+^ epicardial cells that were also positive for CD44 (*SI Appendix*, Fig. S4).

### Expansion of Runx1^+^ cells is driven by myocardial signals

Multiple approaches were taken to find out whether the regulatory role of Dsg2 on cardiogenesis is cardiomyocyte driven.

**Figure 6:**
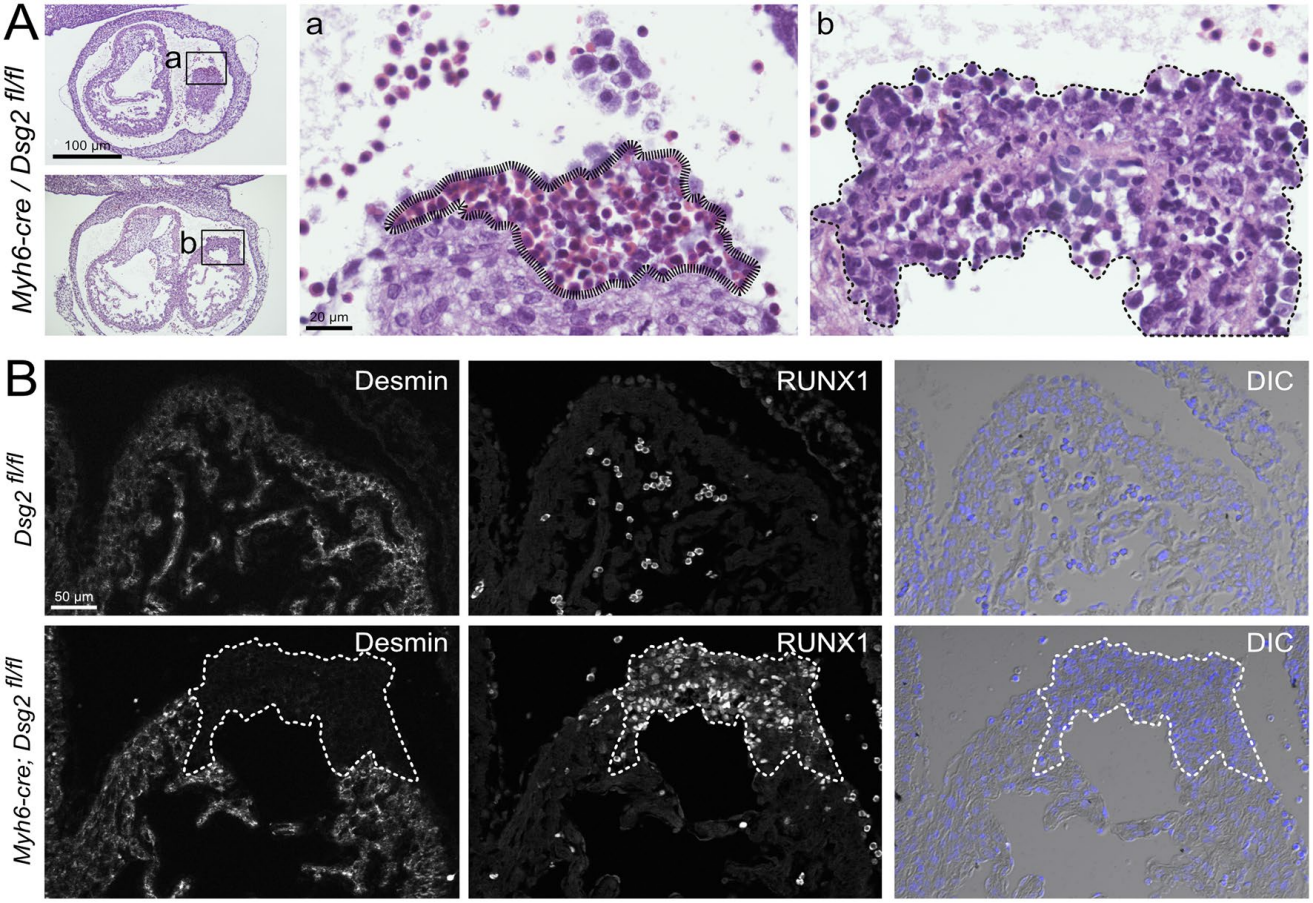
Cardiomyocyte-specific Dsg2 ablation is sufficient for the formation of abnormal type A and type B cell clusters. (**A**) shows photomicrographs of consecutive, hematoxylin eosin-stained sections of a *myh6-cre*^+*/−*^*/Dsg2*^*fl/fl*^ heart with cardiomyocyte-restricted Dsg2 depletion at low and high magnification (boxed region). Note the abnormal clusters of red blood cells in *a* (striated line) and the clusters of ectopic cells on the epicardial and endocardial side in *b* (dashed line). (**B**) The double fluorescence micrographs and corresponding differential interference contrast (DIC) images of control *Dsg2*^*fl/fl*^ and mutant *myh6-cre*^+*/−*^*/Dsg2*^*fl/fl*^ E11.5 myocardium depict loss of desmin^+^ cardiomyocytes and replacement by a Runx1^+^ cell cluster (dashed line). Phenotypic representation of 1 out of 4 *myh6-cre*^+*/−*^*/Dsg2*^*fl/fl*^ hearts. Size bars, 100 µm at left in (**A**) (same magnification in both images); 20 µm in *a* (same magnification in *b*); 50 µm in (**B**) (same magnification in all images).

(i) Immunohistochemistry (*SI Appendix*, Fig. S5) showed that desmosomal Dsg2 expression was strongly reduced in *Dsg2*^*mt/mt*^ hearts. Desmoplakin immunoreactivity was also affected albeit to a much lower degree. Most importantly, punctate anti-Dsg2 immunostaining was completely absent in keratin 8^+^ epicardial and CD31^+^ endocardial cells.

(ii) Cardiac morphogenesis was studied in a cardiomyocyte-specific *Dsg2* knockout line (*myh6-cre*^+*/−*^*/Dsg2*^*fl/fl*^)^4^. Similar to the situation in ubiquitous *Dsg2*^*mt/mt*^ hearts, we were able to identify abnormal type A and type B cell clusters at E11.5 (Fig. 6A). Both cluster types were negative for desmin and, instead, contained an expanding Runx1^+^ cell population (Fig. 6B).

(iii) We investigated, whether endothelial cells in general have an increased capacity of hematopoiesis in the *Dsg2*^*mt/mt*^ background independent of their localization. Runx1 expression in dorsal aorta, which is a major site of hematopoiesis at E10.5 was, however, normal (*SI Appendix*, Fig. S6). Additionally, in an in vitro hematopoietic colony forming assay we could not detect a difference in the number of blood cell colonies obtained from wild-type or *Dsg2*^*mt/mt*^ hearts at E10.5, i.e. a day prior to Runx1^+^ cell expansion of (N = 5 *Dsg2*^*wt*^ and N = 2 *Dsg2*^*mt/mt*^, *p* = 0.8, t-test). Taken together, the experimental evidence strongly suggests that abnormal expansion of Runx1^+^ cells is driven by cardiomyocytes.

### Structural myocardial alterations are associated with altered Notch 1 signaling

**Figure 7:**
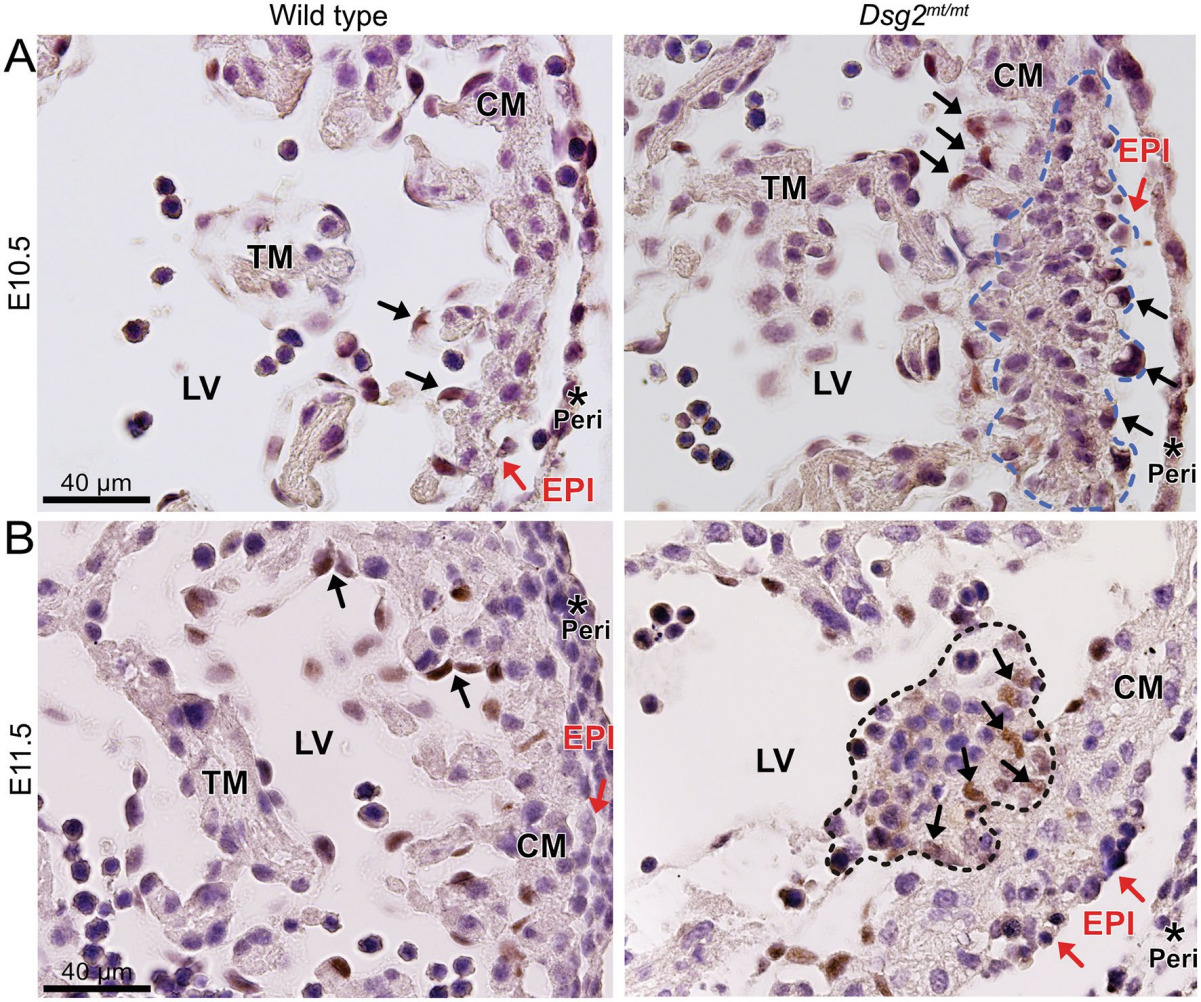
The expression pattern of cleaved Notch1 (N1ICD) is altered in *Dsg2*^*mt/mt*^ embryonic hearts. The images show immunohistological sections of E10.5 (**A**) and E11.5 (**B**) wild-type and Dsg2^mt/mt^ hearts. (**A**) Black arrows point to N1ICD^+^ endocardial cells in the wild-type heart. In contrast, altered-shaped endo and epicardial N1ICD^+^ cells (black arrows) surround disorganized cardiomyocytes (blue dashed line) in *Dsg2*^*mt/mt*^ heart. Data are representative images of three wild type and two *Dsg2*^*mt/mt*^ hearts per group. (**B**) Note, that N1ICD^+^ cells are restricted to scattered endothelial cells in the wild type (black arrows) but are also detectable in *Dsg2*^*mt/mt*^ myocardium (black arrows) harboring a type A cluster (dashed line) next to disarranged cardiomyocytes with mesenchymal-like morphology. LV, left ventricle; RV, right ventricle; TM, trabecular myocardium; CM, compact myocardium; red arrows, epicardium (EPI), *, pericardium (Peri). Data are representative images of wild-type and *Dsg2*^*mt/mt*^ hearts (n = 3 per group). Size bars: 40 μm in (**A**) (same magnification in left and right image) and (**B**) (same magnification in left and right image).

In a search for cardiomyocyte-endocardial signaling that may induce the hematopoietic clusters, we investigated Notch1, which has been shown to be relevant for cardiac morphogenesis and hematopoietic stem cell expansion^31,32^. Notch1 activation is coupled to proteolytic cleavage resulting in cytoplasmic release and nuclear localization of the Notch1 intracellular domain (N1ICD). Immunohistochemical analysis showed that the percentage of N1ICD^+^ endocardial cells did not differ significantly between the wild type and *Dsg2*^*mt/mt*^ at E11.5 (79.9% versus 82.5%; n = 4 for *Dsg2*^*mt/mt*^ and n = 2 for *Dsg2*^*wt/wt*^, *p* = 0.5, t-test; Fig. 7). But an overall increase in N1ICD^+^ cells was detectable in normal-appearing *Dsg2*^*mt/mt*^ myocardium at E11.5 (13% versus 0.5% of N1ICD^+^ cells, respectively; n = 4 for *Dsg2*^*mt/mt*^ and n = 2 for *Dsg2*^*w/wt*^, *p* = 0.02, t-test). Remarkably, N1ICD^+^ cells with an altered shape were detected next to groups of disorganized cardiomyocytes in *Dsg2*^*mt/mt*^ hearts (Fig. 7A). The altered endocardial morphology may indicate incipient endothelial-mesenchymal transition. In support, type A cell clusters were surrounded by N1ICD^+^ cells at E11.5 (Fig. 7B).

**Figure 8:**
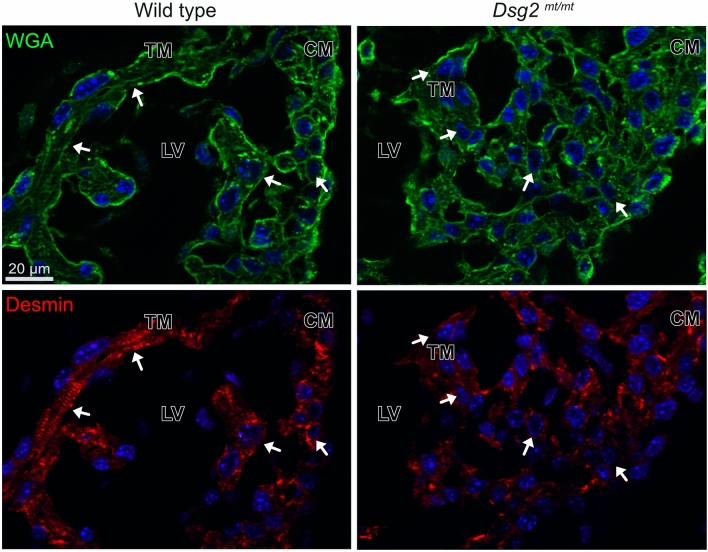
The shape and desmin cytoskeleton of cardiomyocytes is altered in *Dsg2*^*mt/mt*^ myocardium prior to the formation of abnormal cell clusters at E10.5. The double fluorescence microscopy images depict cell borders, that are delineated by fluorescent wheat germ agglutinin (WGA; green), and the desmin cytoskeleton, which is detected by anti-desmin immunofluorescence (red). Arrows in *Dsg2*^*mt/mt*^ point to reduced desmin expression in compact myocardium and loss of sarcomeric distribution in trabecula (compare to white arrows pointing to CM and TM in wild type). LV, left ventricle; TM, trabecular myocardium; CM; compact myocardium. Data are representative images of n = 3–4 hearts per group. Size bar: 20 μm (same magnification in all images).

To substantiate the idea that structural myocardial alterations elicit the pathogenic chain, we examined cell organization at E10.5, when abnormal clusters are not yet formed. Analysis was done by staining cell borders with wheat germ agglutinin (WGA). This revealed the regional disarrangement of multiple layers of cardiomyocytes across compact and trabecular myocardium in *Dsg2*^*mt/mt*^ hearts. Notably, local cardiac tissue disarrangement was prior to and independent of hematopoietic cell/erythrocyte cluster formation (Fig. 8). Anti-desmin staining further revealed reduced desmin expression and loss of sarcomeric organization in these regions (Fig. 8).

## Discussion

### Regulation of cardiogenesis

**Figure 9:**
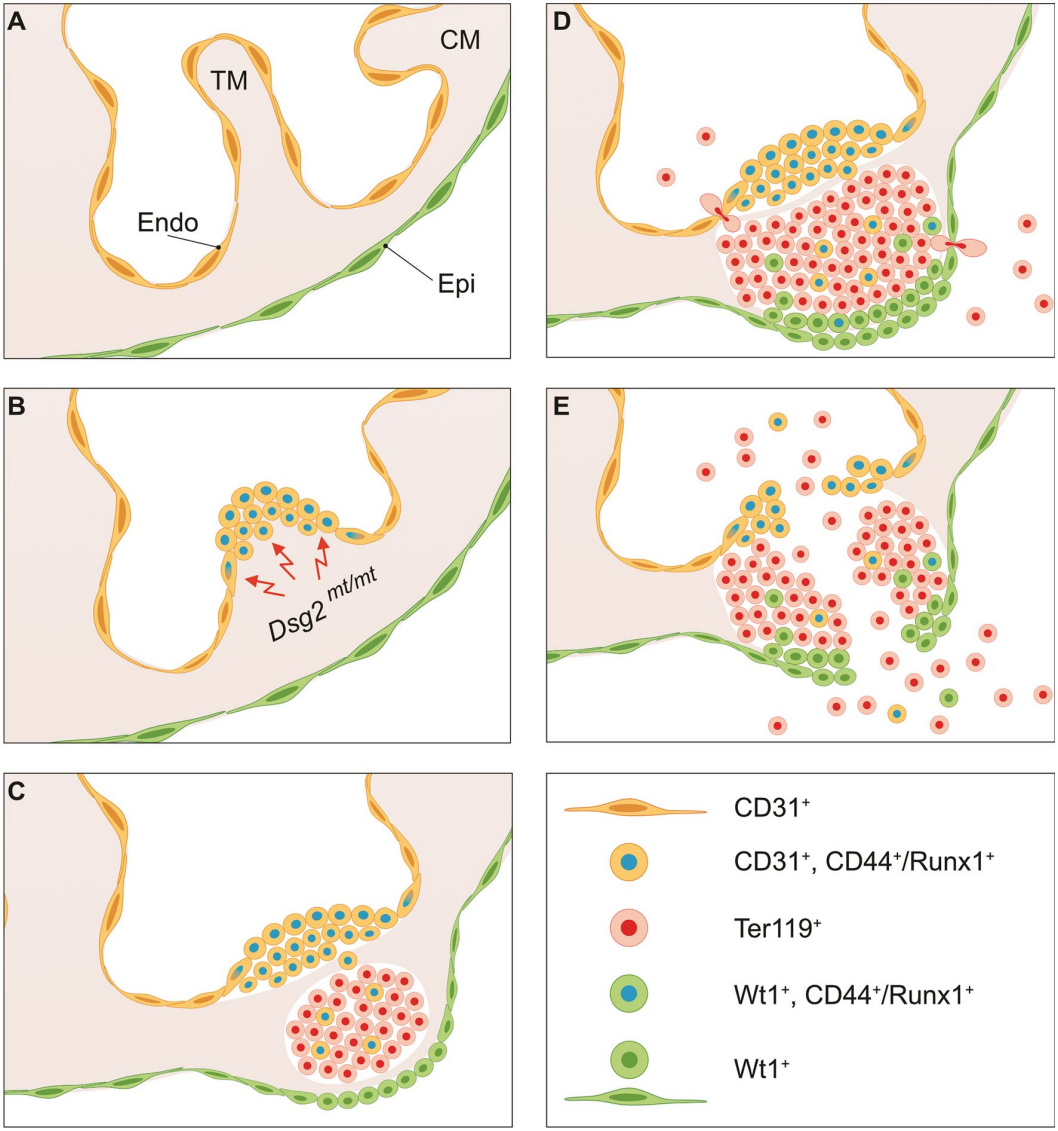
The scheme summarizes findings of altered differentiation in *Dsg2*^*mt/mt*^ embryonic hearts. (**A**) depicts the unperturbed situation in embryonic heart with trabecular and compact myocardium (TM and CM, respectively) that is covered by monolayers of endocardial cells (Endo) facing the heart chamber and epicardial cells (Epi) facing the pericardial cavity. (**B**) In the *Dsg2*^*mt/mt*^ heart loss of cardiomyocyte integrity triggers endocardial CD31^+^ cells to proliferate and differentiate into CD44^+^/Runx1^+^ hematopoietic cells on the tip of trabecular myocardium generating type A cell clusters. (**C**) CD44^+^/Runx1^+^ cells invade the myocardial wall where they differentiate and proliferate forming clusters of Ter119^+^ erythroid type B cell clusters. At the same time, epicardial Wt1^+^ cells obtain a round shape. (**D**) Type A and type B cell clusters proliferate accompanied by increasing numbers of Wt1^+^ cells, some of which become CD44^+^/Runx1^+^ and some of which invade the cell clusters. At the same time, nucleated erythrocytes leave the type B cell clusters by transmigration through endo- and epicardial cells leading to pericardial hemorrhage prior to rupture occurrence. (**E**) Amplification of cells that are less adhesive than cardiomyocytes and concurrent reduction of the myocardial continuity lead to cardiac wall rupture.

The significance of this study is uncovering a novel role of Dsg2 in the regulation of cardiogenesis in the embryonic mouse heart. We demonstrate that mutating *Dsg2* unleashes the differentiation capacity of endocardium and possibly also epicardium to generate Runx1^+^ hematopoietic stem cells leading to the formation of massive accumulations of differentiating erythrocytes during mid gestation. Based on the findings, we propose the following sequence of events (Fig. 9). Dsg2-mutant cardiomyocytes show reduced desmin expression and disorganized myocardium preferentially in the left ventricle and right atrium. This triggers a yet unknown mechanical or paracrine signal. In this way atypical round-shaped CD31^+^ cells are formed in the endocardium with hemogenic potential, which heralds the onset of an intrinsic but usually suppressed differentiation pathway. The unleashed differentiation potential is reflected in the production of Runx1^+^ and CD44^+^ cells. Proliferation of these cells results in the expansion of type A cell clusters, which are in direct continuity with normal-appearing endocardial cells. A possible scenario is that some of the amplified endocardial cells become migratory, loose contact with neighboring cells and invade the myocardium. The loss of cell–cell contact and the altered microenvironment may provide factors that favor the formation of type B cell clusters by inducing erythroid differentiation. The detection of single Runx1^+^/CD44^+^ cells in the type B cell clusters can be taken as evidence for this possibility. Alternatively and simultaneously, epicardial cells may undergo a similar hematopoietic transformation. The local increase in Wt1^+^ epicardial cells, some of which are Runx1^+^ and CD44^+^ supports this possibility. We would, however, like to stress that the hemogenic phenotype of the epicardium was seen only in conjunction with adjacent type A/B cell clusters. These observations suggest that enhanced proliferation and altered differentiation of epicardial cells may rather be a consequence of the formation of type A and type B cell clusters than their cause. Be it as it may, the expansion of the cell clusters, especially of the type B cell clusters, disrupts myocardial connectivity. The developing erythrocytes are much less adhesive than the tightly connected cardiomyocytes. Once the cell clusters reach full wall thickness, cardiac contractility may exert sufficient strain to disrupt the dysfunctional myocardial wall leading to heart rupture with pericardial hemorrhage. Lethal heart rupture, however, did not occur in all mutants (see also^3^). Several E11.5–12.5 mutant embryos repair their heart muscle without any remaining damage. This may, at least in part, be due to extrusion of abnormal cell clusters and transmigration of matured erythrocytes from type B lesions either into the heart chambers or the pericardial cavity. Interestingly, mid gestation embryonic mortality has also been reported in mice with null mutation in the desmosomal proteins plakoglobin (Jup) and plakophilin 2 (Pkp2)^13,33^. Similar to the *Dsg2*^*mt/mt*^ embryos, pericardial hemorrhage and reduced trabeculation was observed. Notably, reduced Dsg2 expression was reported in Pkp2-deficient hearts, suggesting they may have similar mechanisms in the dysregulation of cardiogenesis.

### Endocardial to hematopoietic transition

An important event in the *Dsg2*^*mt/mt*^ myocardium is the endocardial to hematopoietic transition, which is sporadic by nature in the normal embryonic heart^24^. It is well documented that mechanical forces generated by blood flow induce shear and frictional stress parallel to endothelial cells, which respond by expressing signaling molecules and hematopoietic master regulators, including Runx1, and thereby induce the formation of hematopoietic cell colonies^34–36^. Altered biomechanical coupling and sensing may lead to the observed increased endothelial transformation to hematopoietic cells in the *Dsg2*^*mt/mt*^ endocardium. In support, a biomechanical function has been demonstrated for Dsg2 in cardiomyocytes and in experiments using Dsg2-specific or other desmosome-specific tension sensors^37–41^. Furthermore, altered response to shear stress has been reported to occur in a cardiomyocyte model of arrhythmogenic cardiomyopathy^42^. The altered cardiomyocyte shape and perturbed sarcomere organization may elicit similar responses by changing the cardiac force balance in the Dsg2-mutant embryonic heart. Another pathogenic mechanism may be paracrine signaling, which has been shown to regulate skin pigmentation in situations of altered desmoglein 1 expression^43^ and very recently also for epicardial signaling in adult murine, desmoplakin-linked AC^44^. The observation that cardiomyocyte-specific Dsg2 deletion is sufficient to induce the formation of type A and type B cell clusters lends strong support to altered paracrine signaling in our mouse model, whereby the hematopoetic differentiation potential of endocardial cells is unleashed.

### Expansion of Runx1^+^ cells

Expansion of Runx1^+^ cell clusters is the fundamental event that disrupts cardiogenesis in *Dsg2* mutant hearts. Apart from the function of Runx1 in hematopoietic stem cell differentiation^45^, it is associated with cardiomyopathy in the adult heart^46^. Its role in cardiac biology, however, is controversial: Histological studies suggested that Runx1 is a marker of dedifferentiating cardiomyocytes^47,48^, whereas a recent single cell transcriptomic analysis of human heart suggested that Runx1 is a regulator of myofibroblast differentiation after myocardial infarction^49^. Despite this ambiguity, Runx1 deficiency protects the injured murine heart from adverse cardiac remodeling^50^. In our study, Runx1^+^ cells were found in replacements of compact myocardium that were devoid of cardiomyocyte-specific markers (fetal cardiac actin, desmin, β-catenin and desmoplakin). In addition, a subset of Wt1^+^ and CD31^+^ cells expressed Runx1. Since fibroblasts are completely absent in the myocardium until E12.5, Runx1 activation induces hematopoietic cell differentiation in E10.5/11.5 hearts and does not activate fibrotic pathways as is the case in the adult heart^49^.

### Notch1 signaling

Our findings of N1ICD^+^ cells in the *Dsg2*^*mt/mt*^ myocardium either next to or independent of abnormal cell clusters point to a possible link between structural cardiomyocyte alterations and endocardial transdifferentiation. It is known that Notch1 signaling is important for cardiomyocyte-endocardial cross-talk, trabecular myocardial growth and the generation of hematopoietic cells from endothelial cells^31,32^. It may also be of relevance that epicardial N1ICD activation has been shown to induce a phenotype with pericardial bleeding and subepicardial erythrocyte expansion that is very similar to that observed in Dsg2-mutant embryos^51^. The regulation of Notch1 signaling is complicated by action of multiple ligands that induce different cell fates (i.e., Jagged and Delta). Importantly, it has been demonstrated that the strength of Notch1 signaling is affected by hemodynamic stress involving the interaction of intermediate filaments with Jagged 1^52^. The reduced Dsg2 expression in conjunction with the altered desmin cytoskeleton in both *Dsg2*-mutant strains may thus provide the myocardial stimulus for the induction of Notch1 signaling. We suggest, that Notch1 signaling in *Dsg2*^*mt/mt*^ hearts is redirected from its normal function of supporting trabecular growth to instruct endocardial transdifferentiation instead. The exact molecular mechanism that links loss of myocardial Dsg2 to transdifferentiation of endocardial cells is yet to be discovered.

## Materials and methods

### Animals

*Dsg2 *^*cKO*^* (Myh6-Cre*^+^*/Dsg2*^*fl/fl*^*)* mice with cardiomyocyte specific *Dsg2* ablation and *Dsg2*^*mt/mt*^ mice lacking exons 4 to 6 of the *Dsg2* gene have been described^3, 4^. *Dsg2*^*wt/mt*^ females were bred with *Dsg2*^*wt/mt*^ or *Dsg2*^*mt/mt*^ males to obtain *Dsg2*^*mt/mt*^ homozygous mutant embryos. *Dsg2*^*wt/wt*^ and *Dsg2*^*wt/mt*^ littermates or independently generated, age-matched embryos of our mouse colony were used as control. Females were checked for mating plugs and the morning of plug positivity was counted as embryonic day E0.5. Embryos were harvested and genotyped at E10.5, E11.5, E12.5 and E14.5. In the conditional knock out line, *Dsg2*^*fl/fl*^ females were bred with *Myh6-Cre*^+^*/Dsg2*^*fl/fl*^ males and embryos were assessed at E11.5. All animal procedures were performed in accordance with the recommendations in the Guide for the Care and Use of Laboratory Animals. Animal experiments were approved by the Ministry for Environment, Agriculture, Conservation and Consumer Protection (LANUV) of the State of North Rhine-Westphalia, Germany (84.02.04.2013.A145 and 84.02.04.2015.A190 and A4 notifications for killing animals for scientific purposes). The study is reported in accordance with ARRIVE guidelines.

### Genotyping

For genotyping of weaned animals ear tissue obtained from ear punching was used; for genotyping of embryos parts of the lower body or the head were used. The tissue was dissolved in 250 µl 50 mM NaOH while shaking at 95 °C for one hour. After cooling on ice for 2 min, 25 µl of TE buffer (1 M Tris/HCl, 10 mM EDTA, pH 8) was added and 1 µl of the solution was taken for subsequent PCR analyses. A combination of three primers (*Dsg2*^*mt/wt*^ forward primer: GTCATCCACAGCCTCATAAC; *Dsg2*^*wt*^ reverse primer AAGTGCATCCTCCTCTAC; *Dsg2*^*mt*^ reverse primer CTTCCACCGTCAAGGAATAG) was used to detect the *Dsg2*^*wt*^ and *Dsg2*^*mt*^ alleles (product size of 289 bp and 434 bp, respectively). *Dsg2*^*fl*^ was detected by the following primers: forward GGTAAATGCAGACGGATCAG and reverse TGGGCTACACTCATAGGAAG (product size 117 bps for WT and 167 for *Dsg2*^*fl*^). *Myh6-cre* was detected with forward primer ATGACAGACAGATCCCTCCTATCTCC and reverse primer CTCATCACTCGTTGCATCATCGAC (product size 300 bps). Successful *myh6-cre*-mediated recombination and *Dsg2* knockout in embryonic heart was confirmed by anti-Dsg2 immunofluorescence^4^.

### Histology, immunofluorescence, immunohistochemistry and microscopy

Embryos were fixed in freshly-prepared 4% (w/v) formaldehyde in PBS overnight, dehydrated in isopropanol and embedded in paraffin. Serial 5 μm-thick sections were prepared and stained with hematoxylin/eosin or used for immunoanalysis. For immunofluorescence labelling and immunohistochemistry, tissue sections were deparaffinized, rehydrated and antigen retrieval was accomplished by cooking the slides in 10 mM citrate buffer (pH 6) in a pressure cooker at 121 °C for 3 min). Sections were then incubated overnight with primary antibody diluted in 1.5% (w/v) bovine serum albumin (BSA) in PBS buffer at 4 °C, after which they were washed three times in Tris/HCl buffer (pH 7.5). For immunofluorescence staining, sections were then incubated with secondary antibodies for one hour at room temperature and were washed three times. Nuclear staining was done by incubation in PBS with 2 μg/ml 4',6-diamidin-2-phenylindol (DAPI) for 30 min prior to mounting. For immunohistochemistry color detection of the primary rat and rabbit antibodies was done using the Histofine Simple Stain Mouse MAX PO (Rat)-Kit and the ZytoChem Plus (HRP) Polymer Kit, respectively, following the protocol provided by the manufacturers followed by 3,3'-Diaminobenzidin (DAB) staining and hematoxylin counterstaining. A complete list of antibodies and their specific dilutions is provided in Table S2. For visualization of cell border tissue sections were incubated with tetramethylrhodamine isothiocyanate-labeled wheat germ agglutinin (100 µg/ml, ThermoFisher) for 30 min at room temperature.

Light and fluorescence microscopic images were recorded with Axiophot and ApoTome.2 imaging systems (both from Zeiss) and processed by Zen 2 and Zen 3.3 (blue edition; https://www.zeiss.com/microscopy/int/products/microscope-software/zen.html ), respectively.

Three dimensional reconstruction of embryonic hearts was done with the help of Amira software (version 6.2; https://www.thermofisher.com/de/de/home/electron-microscopy/products/software-em-3d-vis/amira-software.html). Hearts were serially sectioned, stained with hematoxylin/eosin and imaged. Images were aligned and each slice was labeled accordingly using the segmentation tool. Reconstruction was completed by generation of a surface view.

### Colony forming assay

Embryonic hearts were dissected at E10.5, washed in PBS and digested in 2.5 µg/ml collagenase (Sigma) for 30 min at 37 °C. This was followed by mechanical dissociation and filtering through a 70 µm cell strainer (Corning). Cells of each heart were counted and seeded with methylcellulose complete media (HSC007, R&D) in 24-well plates at 37 °C with 5% CO_2_ for 2 weeks. Afterward the resulting colonies were characterized and counted. The number of colonies were normalized based on the initial seeding number.

### Electron microscopy

Electron microscopy was done as previously described^4,5^. In brief, embryonic hearts were excised and directly fixed in 4% formaldehyde/1% glutaraldehyde for 2 h. Remaining embryonic tissue was used for genotyping. The formaldehyde/glutaraldehyde-fixed heart samples were subsequently washed in 0.1 M phosphate buffer (pH 7.2–7.4) and were treated with 0.5% uranylacetate in 0.05 M sodium maleate buffer (pH 5.2) for 2 h in the dark. The tissue was dehydrated and embedded in araldite using acetone as intermedium. Polymerization was carried out at 60 °C for 48 h. 0.35 µm semithin sections were prepared with an ultramicrotome and stained with toluidine blue. Ultrathin sections of suitable regions were then prepared. To enhance contrast, the sections were first treated with 3% uranyl acetate for 5 min and then with 0.08 M lead citrate solution for 4 min. Pictures were taken on an EM 10 (Zeiss) with a digital camera (Olympus) using the iTEM software (Olympus; https://resaltatech.com/item_platform_main.htm).

## Supplementary Information

Supplementary Information.
