# Supplementary material for: Desmoglein 2 regulates cardiogenesis by restricting hematopoiesis in the developing murine heart
Source: Sci Rep. 2021 Nov 4;11:21687. doi: 10.1038/s41598-021-00996-y (PMC8569146; doi:10.1038/s41598-021-00996-y)
Supplement: Supplementary file 1 — Supplementary Information. [file 41598_2021_996_MOESM1_ESM.docx]

**Supplementary information:**

**Desmoglein 2 regulates cardiogenesis by restricting hematopoiesis in the developing murine heart**

Hoda Moazzen , Kateryna Venger, Sebastian Kant, Rudolf E. Leube & Claudia A. Krusche

Figures S1 to S6

Tables S1 to S2


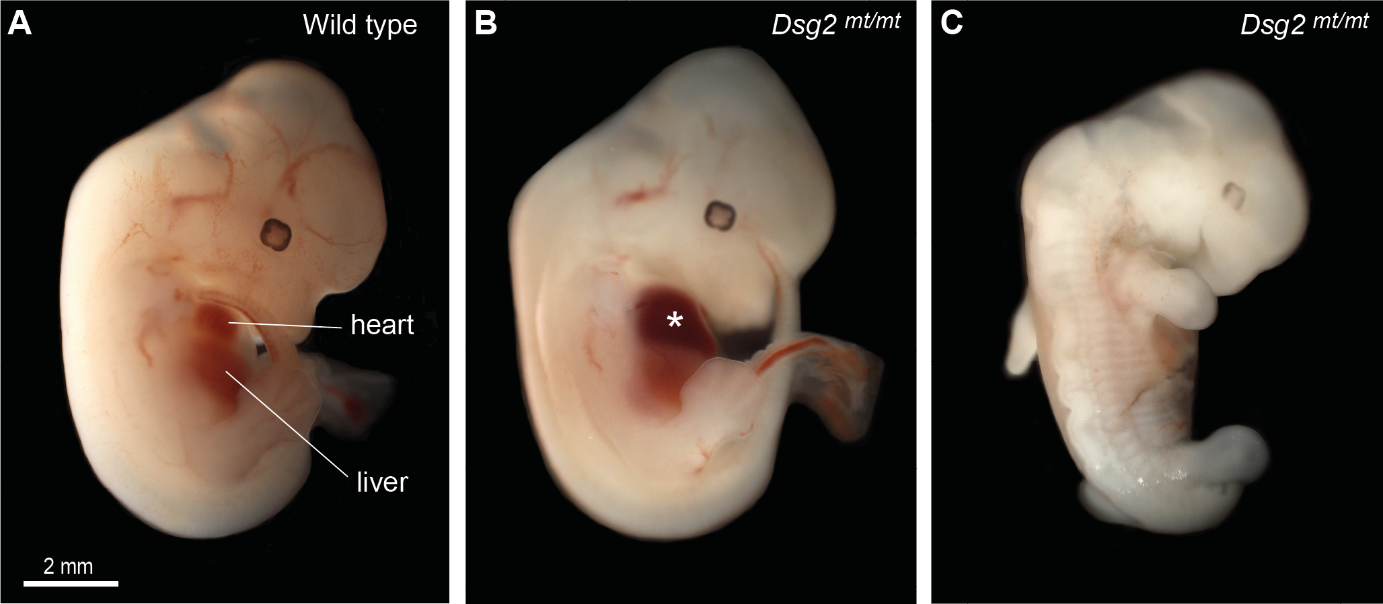


**Fig. S1.** E12.5 *Dsg2^mt/mt^* embryos suffer from pericardial hemorrhage and some die. The photographs show a comparison of a dissected wild-type embryo (*A*) with two dissected *Dsg2^mt/mt^* embryos (*B, C*). Note the pericardial hemorrhage in (*B, **) and the overall atrophy in (*C*).

**
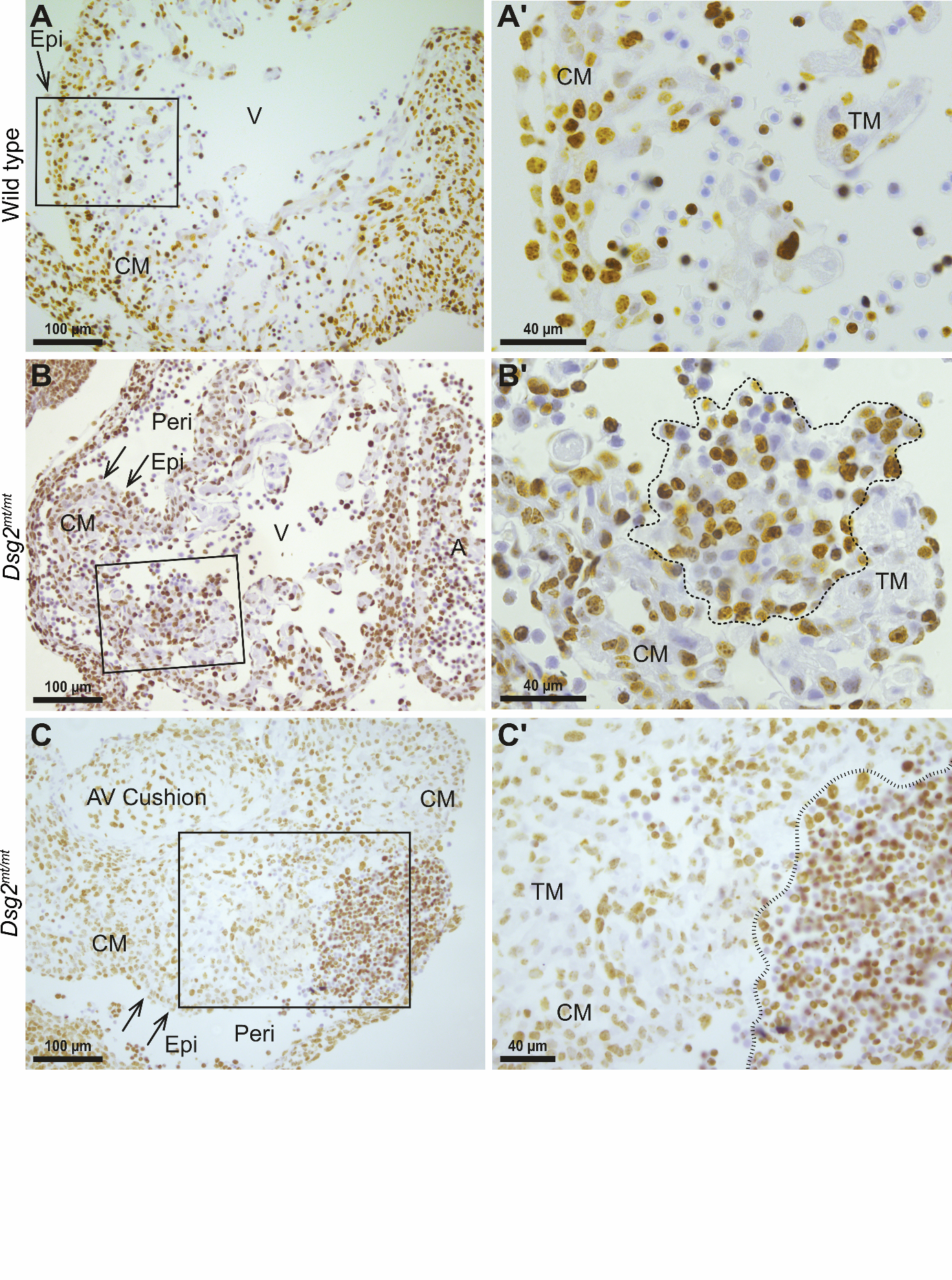
**

**Fig. S2.** Immunohistochemical detection of Ki67 antigen (brown) reveals proliferating cells in wild-type *(A, A’)* and *Dsg2^mt/mt^* heart *(B-C)* at E11.5*.* The endocard-associated type A (*B, B'*; dashed line) and subepicardial type B (*C, C'*; striated line) cell clusters of *Dsg2^mt/mt^* hearts contain many Ki67^+^ cells. The boxed areas in (*A*) to (*C*) are shown at higher magnification in (*A*') to (*C* '), respectively. Data are representative images of n = 3-4 hearts per group. Peri, pericardial cavity; Epi, epicardium (arrows); V, ventricle; AV cushion, atrioventricular cushion; TM, trabecular myocardium; CM, compact myocardium. Size bars: 100 μm in *A* to *C* and 40 μm in *A' to C'.*


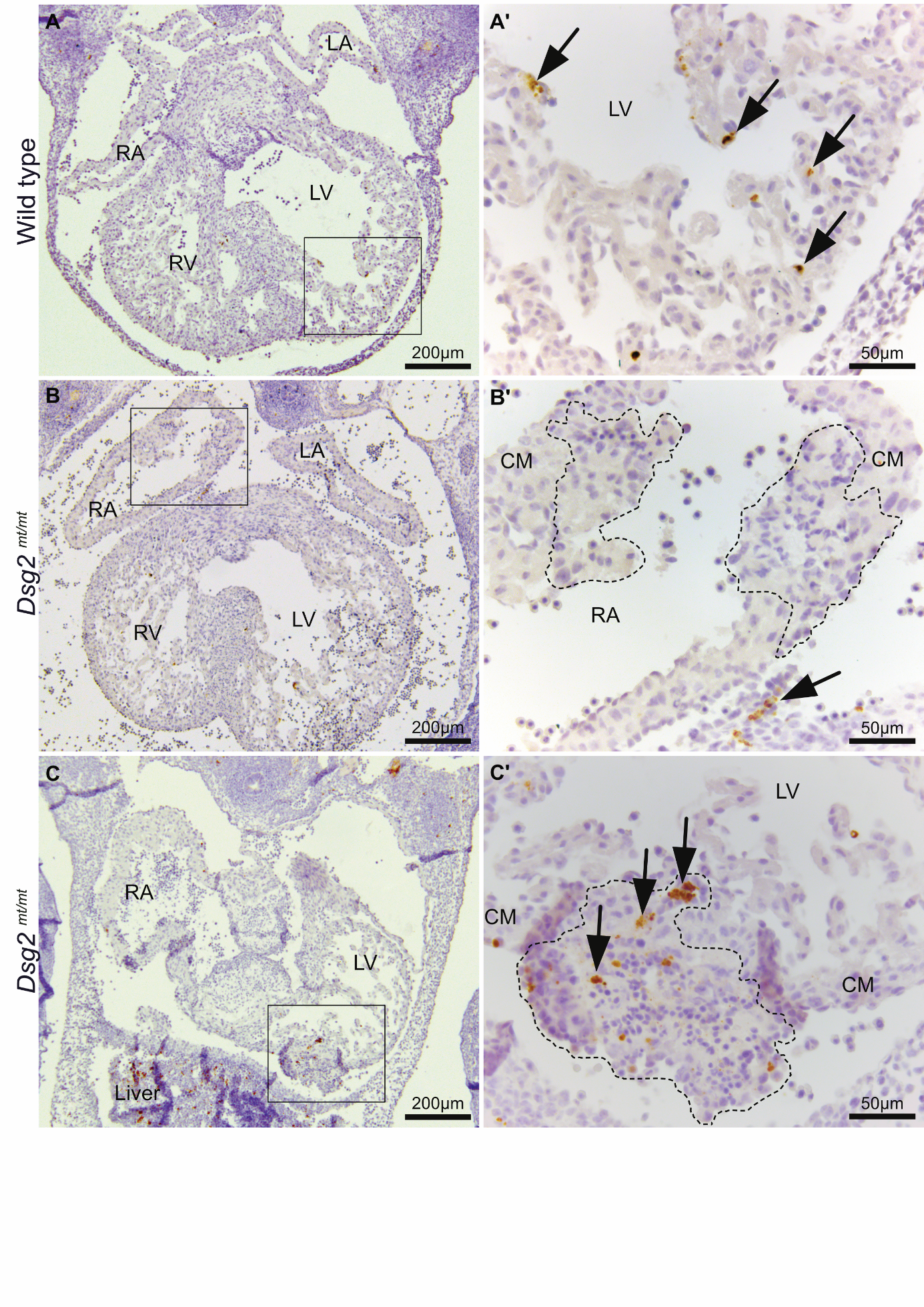


**Fig. S3.** *Dsg2^mt/mt^* cardiomyocytes in contact with expanding cell clusters do not undergo apoptosis. Immunostaining for cleaved caspase-3 marks apoptotic cells (brown; arrows) in wild-type (*A, A’*) and *Dsg2^mt/mt^* hearts (*B-C’*) at E11.5. Dashed lines delineate regions of disorganized cells in *B’* and a type A cluster in *C’*. Apoptotic cells are seen within the expelled cells in the pericardium (*B’*) or in the expanding cell cluster (*C’*). The boxed areas in (*A)* to (*C)* are shown at higher magnification in (*A*') to (*C* '), respectively. The data are representative images of n = 3-4 embryonic hearts per group. CM, compact myocardium; LV, left ventricle, RA, right atrium. Size bars: 200 μm in *A* to *C* and 50 μm in *A' to C'.*


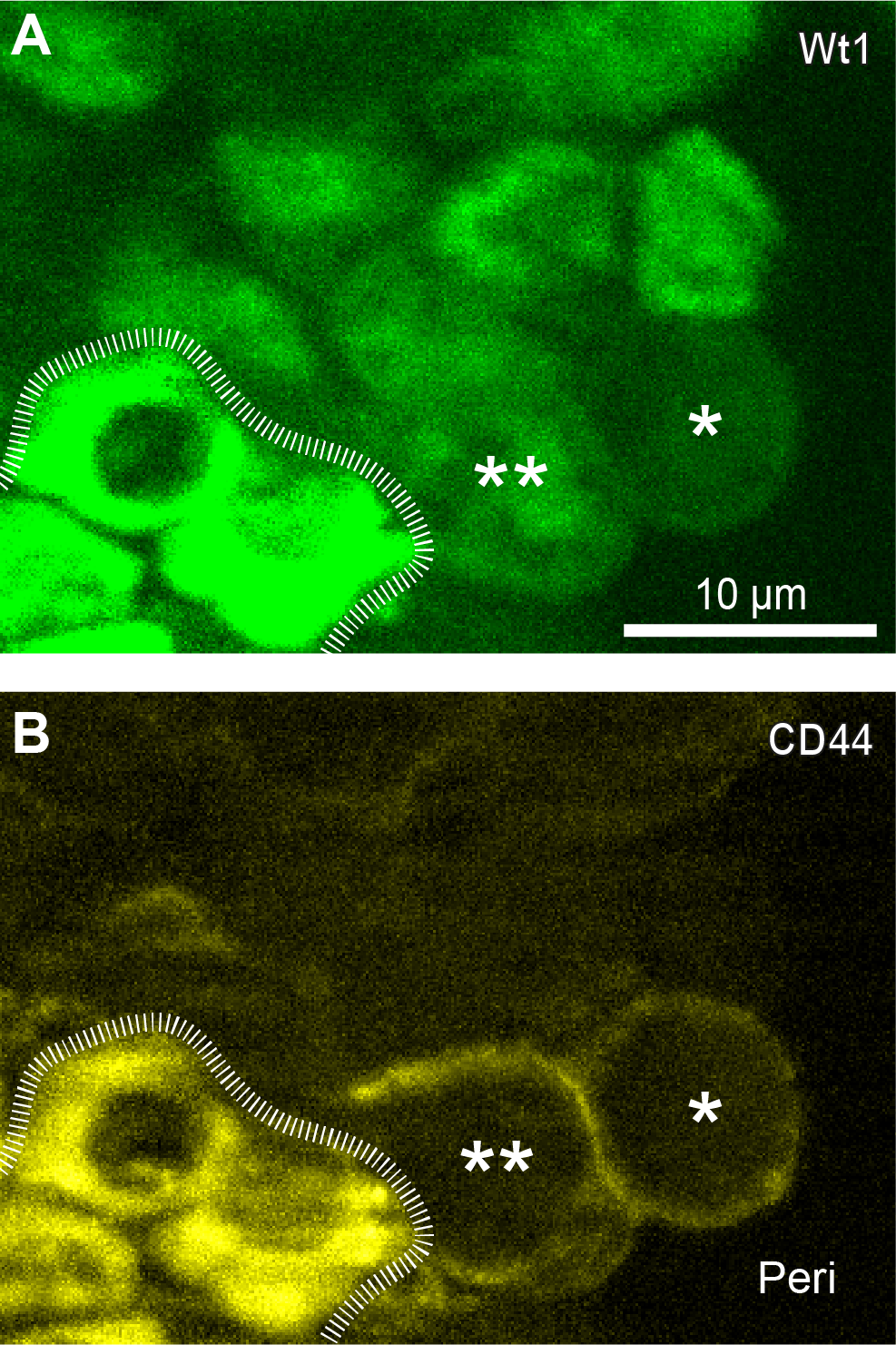


**Fig. S4.** Wt1^+^/CD44^+^ cells are rarely detectable in *Dsg2^mt/mt^* embryos. The fluorescence micrograph in (*A)* depicts a group of atypically round-shaped Wt1^+^ cells that are next to a type B cell cluster with highly autofluorescent developing erythrocytes (demarcated by striated line) of E12.5 embryonic heart. Co-localization of anti-CD44 antibodies (*B*) identifies a rare Wt1^+^/CD44^+^ cell (**) next to a Wt1^-^/CD44^+^ (*) cell and multiple Wt1^+^/CD44^-^ cells (not marked). Data are representative images of n = 2-3 hearts per group. Peri, pericardium, Size bar, 10 µm in *A* (same magnification in *B*).

**
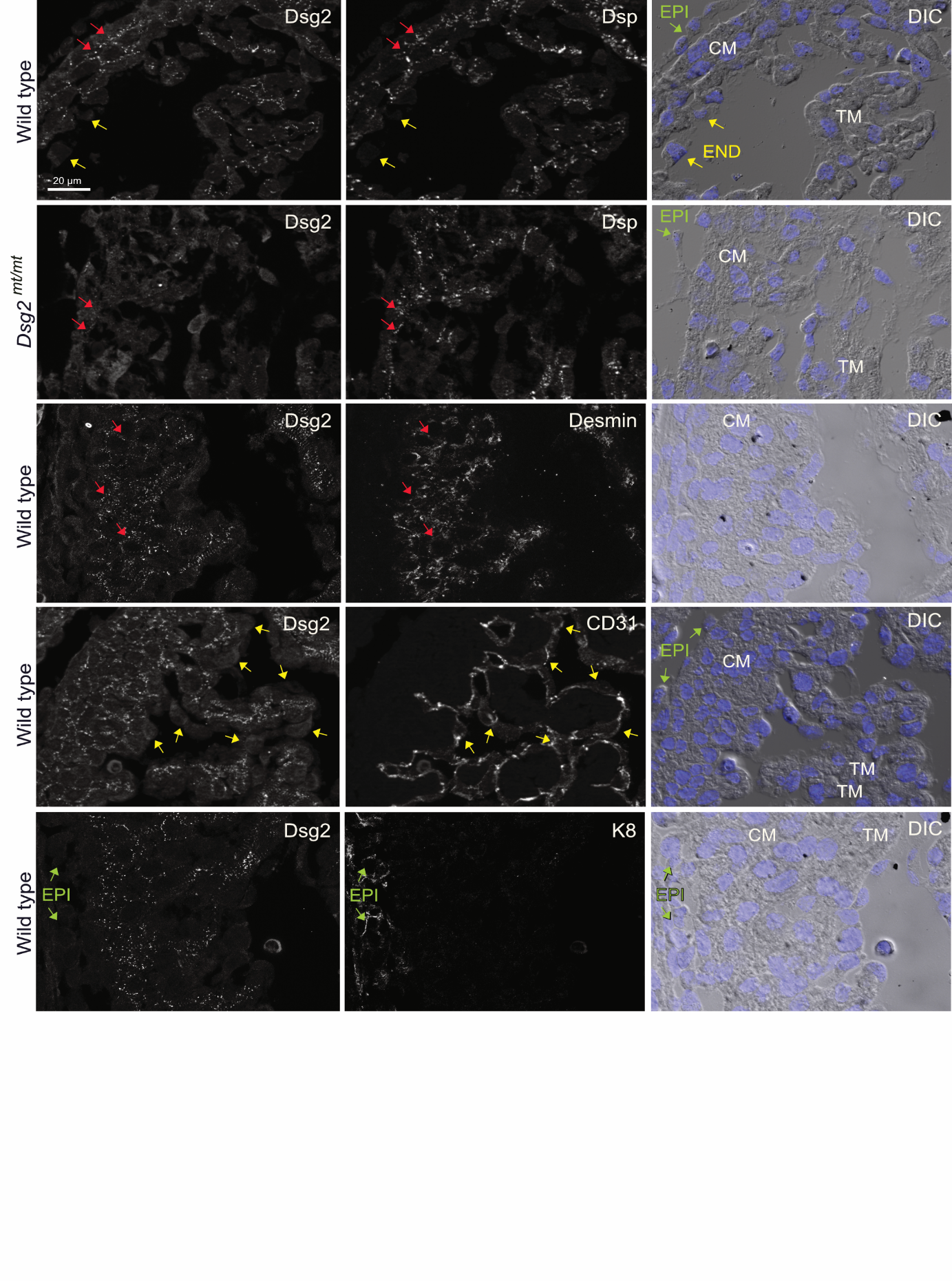
**

**Fig. S5.** Desmosomal Dsg2 expression is restricted to the myocardium. The microscopic pictures show double immunofluorescence recordings detecting Dsg2 together with a marker for desmosomes (desmoplakin; Dsp), myocardium (desmin), endocard (CD31) and epicard (keratin 8; K8) in wild-type and *Dsg2^mt/mt^* E11.5 hearts. Corresponding differential interference contrast (DIC) and nuclear DAPI stains are shown in the right panel. Note, that Dsg2 co-localizes with desmoplakin in distinct puncta, that Dsg2 is co-expressed with desmin in cardiomyocytes (red arrows) but is absent in CD31^+^ endocardial cells (END; yellow arrows) and keratin 8^+^ epicardial cells (EPI; green arrows). Data are representative images of n = 3 hearts per group. TM, trabecular myocardium; CM, compact myocardium. Size bars, 20 µm (same magnification in all images).


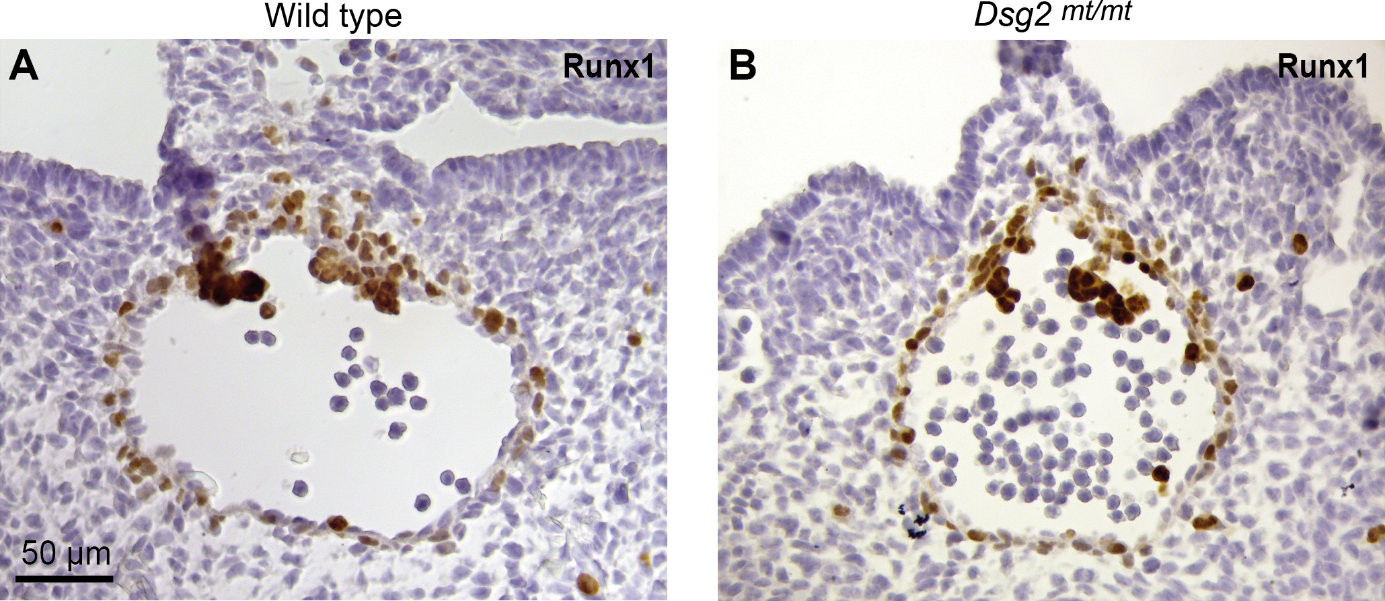


**Fig. S6.** Runx1 expression in the dorsal aorta does not differ between *Dsg2^mt/mt^* and wild-type E10.5 embryos. The images show Runx1 immunoreactivity. Data are representative images of n = 4 hearts per group. Size bar, 50 µm in *A* (same magnification in *B*).

**Supplementary Tables**

**Table S1.** Characterization and prevalence of cardiovascular abnormalities among *Dsg2^mt/mt^* embryos. None of the listed abnormalities were identified in control mice.

|  | E11.5 | E12.5 | E14.5 |  |
| --- | --- | --- | --- | --- |
|  | N (%) | N (%) | N (%) |  |
| Total number of embryos | 24 (100.0) | 12 (100) | 12 (100.0) |  |
| - Dead embryos | 0 (0) | 4 (33.3) | 4 (33.3) |  |
| - Vital embryos | 24 (100.0) | 8 (66.7) | 8 (66.7) |  |
| - Normal | 6 (25) | 2 (25) | 0 (0) |  |
| - Abnormal | 18 (75) | 6 (75) | 8 (100.0) |  |
| - Pericardial hemorrhage | 15 (83.3) | 6 (100) | 7 (87.5) |  |
| - Type A cell clusters | 16 (88.9) | 4 (66.6) | 6 (75.0) |  |
| - Type B cell clusters | 7 (38.9) | 1 (16.6) | 5 (62.5) |  |
| - Rupture | 2 (11.1) | 1 (16.6) | 4 (50.0) | |

**Table S2.** List of primary and secondary antibodies used in the study.

| **Primary Antibody** | **Host Species** | **Dilution** | **Supplier (Ordering Number)** | **Secondary Antibody (Label)/detection kit** |
| --- | --- | --- | --- | --- |
| Anti-fetal cardiac actin (clone Ac1-20.4.2) | Mouse | 1:50 | Progen (61075) | Anti-mouse IgG (Alexa 647) |
| Anti-desmin | Mouse | 1:1000 | Sigma (SAB4200707) | Anti-mouse IgG (Alexa 647) |
| Anti-Wilms tumor 1 | Rabbit | 1:500 | Abcam (CA 1026) | Anti-rabbit IgG (Alexa 488) |
| Anti-Desmoglein 2 | Rabbit | 1:500 | Own production* | Anti-rabbit IgG (Alexa 488) |
| Anti-Desmoplakin 1 | Guinea pig | 1:1000 | Progen | Anti-guinea pig IgG (Alexa 555) |
| Anti-N-cadherin | Rabbit | 1:500 | Abcam | Anti-rabbit IgG (Alexa 488) |
| Anti-β-catenin | Rabbit | 1:500 | Sigma | Zytochem Plus kit (HRP) Polymer Kit |
| Anti-CD31 (clone SZ31) | Rat | 1:200 | Dianova (DIA-310) | Anti-rat IgG (Alexa 555) |
| Anti-CD44 | Rat | 1:500 | BD Pharmingen  (550538) | Anti-rat IgG (Alexa 555)  Or Simple Stain Mouse MAX PO (anti-rat) |
| Anti-Runx1 | Rabbit | 1:500 | Abcam (ab92336) | Zytomed Plus kit (HRP) Polymer Kit or Anti-rabbit IgG (Alexa 488) |
| Anti-Ter119 | Rat | 1:200 | STEMCELL Technologies (60033) | Simple Stain Mouse MAX PO (anti-rat) |
| Anti- Notch1 (cleaved) | Rabbit | 1:100 | Cell Signaling Technology | Zytomed Plus kit (HRP) Polymer Kit |
| Anti-Ki67 (clone SP6) | Rabbit | 1:200 | Zytomed Systems | Zytomed Plus kit (HRP) Polymer Kit |
| Anti-Keratin 8 | Rat | 1:10 | Developmental Studies Hybridoma Bank (TROMA-I) | Anti-rat IgG (Alexa 555) |
| Anti-cleaved caspase-3 | Rabbit | 1:300 | Zytomed Systems | Zytomed Plus kit (HRP) Polymer Kit |

* N. Schlegel et al., Desmoglein 2-mediated adhesion is required for intestinal epithelial barrier integrity. Am J Physiol Gastrointest Liver Physiol 298, G774-783 (2010)
